# Diagnostic accuracy of contrast-enhanced diffusion-weighted MRI for liver metastases of pancreatic cancer: towards adequate staging and follow-up of pancreatic cancer – DIA-PANC study: study protocol for an international, multicenter, diagnostic trial

**DOI:** 10.1186/s12885-020-07226-0

**Published:** 2020-08-10

**Authors:** G. Litjens, D. M. Rivière, E. J. M. van Geenen, S. A. Radema, L. A. A. Brosens, M. Prokop, C. J. H. M. van Laarhoven, J. J. Hermans

**Affiliations:** 1 Department of Radiology and Nuclear Medicine, Radboudumc, Nijmegen, The Netherlands; 2 Department of Gastroenterology and Hepatology, Radboudumc, Nijmegen, The Netherlands; 3 Department of Medical Oncology, Radboudumc, Nijmegen, The Netherlands; 4 Department of Pathology, Radboudumc, Nijmegen, The Netherlands; 5 Department of Pathology, University Medical Center, Utrecht, The Netherlands; 6 Department of Surgery, Radboudumc, Nijmegen, The Netherlands

**Keywords:** Pancreatic cancer, Liver metastases, MRI, Staging

## Abstract

**Background:**

At the time of surgery, approximately 10–20% of the patients with pancreatic cancer are considered unresectable because of unexpected liver metastasis, peritoneal carcinomatosis or locally advanced disease. This leads to futile surgical treatment with all the associated morbidity, mortality and costs. More than 50% of all liver metastases develop in the first six months postoperatively. These (subcentimeter) liver metastases are most likely already present at the time of diagnosis and have not been identified pre-operatively, due to the poor sensitivity of routine preoperative contrast-enhanced CT (CECT).

**Methods:**

The DIA-PANC study is a prospective, international, multicenter, diagnostic cohort study investigating diffusion-weighted, contrast-enhanced MRI for the detection of liver metastases in patients with all stages of pancreatic cancer. Indeterminate or malignant liver lesions on MRI will be further investigated histopathologically. For patients with suspected liver lesions without histopathological proof, follow up imaging with paired CT and MRI at 3-, 6- and 12-months will serve as an alternative reference standard.

**Discussion:**

The DIA-PANC trial is expected to report high-level evidence of the diagnostic accuracy of MRI for the detection of liver metastases, resulting in significant value for clinical decision making, guideline development and improved stratification for treatment strategies and future trials. Furthermore, DIA-PANC will contribute to our knowledge of liver metastases regarding incidence, imaging characteristics, their number and extent, and their change in time with or without treatment. It will enhance the worldwide implementation of MRI and consequently improve personalized treatment of patients with suspected pancreatic ductal adenocarcinoma.

**Trial registration:**

ClinicalTrials.gov Identifier: NCT03469726. Registered on March 19th 2018 - Retrospectively registered.

## Background

Pancreatic ductal adenocarcinoma (PDAC) is one of the most lethal forms of cancer and expected to become the second leading cause of cancer-related deaths before 2030. Developments in pancreatic cancer diagnostics, surgical techniques and treatment have hardly improved the survival rate in the past 40 years. The 5-year relative survival rate as reported by the American Cancer Society remains only 8% [1, 2].

Only 5–25% of all patients are eligible for surgery, to date the only potential cure [3]. Approximately 40–45% of all patients with pancreatic cancer have metastatic disease at diagnosis and 40% of all patients have locally advanced disease with tumor involvement of surrounding vessels or organs. At the time of surgery, approximately 10–20% of the patients are considered unresectable because of unexpected liver metastasis, peritoneal carcinomatosis or locally advanced disease [4–6].

More than 50% of all liver metastases develop in the first six months postoperatively [7]. These liver metastases are most likely already present at the time of diagnosis and have not been identified pre-operatively, as they are too small to be detected by routine preoperative ultrasound and contrast-enhanced CT (CECT) [8, 9].

CECT is highly accurate in assessing the relationship of the tumor to critical arterial and venous structures, since their involvement can preclude surgical resection. However, CECT has a poor sensitivity (38–76%) for the detection and characterization of liver metastases [7, 10–13], especially for subcentimeter metastases, which are often present in pancreatic cancer [14]. This leads to futile surgical treatment with all the associated morbidity, mortality and costs. Moreover, patients who were explored with curative intent and were found unresectable due to peritoneal or liver metastases had a worse overall survival compared to patients with unexpected locally advanced disease [15].

Nowadays, diffusion-weighted MR imaging (DWI) appears to be valuable in both detection and characterization of focal liver lesions with a high sensitivity (86–97%), even for subcentimeter lesions (60–91%) [16–18]. This technique can be used to detect and characterize liver lesions based on decreased diffusion of water molecules caused by tumoral hypercellularity and reduced extracellular space. DWI is especially useful for detecting subcentimeter liver metastases, it is more accurate than conventional T2-weighted imaging techniques, because signal suppression of intravascular flow is obtained (black blood effect) while maintaining good residual signal of the liver lesions [19]. It is easy to implement and adds very little time to a standard MRI examination. However, without high-quality evidence of the benefit of MRI, the use of MRI as part of the routine workup is questioned and therefore not implemented. Currently most guidelines advise to use MRI as a problem-solving tool in addition to CECT; e.g. when the primary tumor cannot be visualized, or in case of undefined liver lesions [20–22]. The American Society of Clinical Oncology (ASCO) leaves the choice of imaging modality in the hands of the physician [23]. MRI is advised for all patients according to the Japanese guideline; however, the level of evidence is low (grade C) [24].

Most studies that have been performed for liver metastases of PDAC are retrospective, including our single center study in patients with potentially resectable pancreatic cancer without liver metastases on CECT [25]. In this study Gadolinium (Gd) enhanced MRI with DWI detected synchronous liver metastases in 24% of patients with potentially resectable pancreatic cancer on CECT with a sensitivity of 84%. DWI showed more lesions than Gd-enhanced MRI, most of which were particularly small (< 5 mm). Correspondingly, the only prospective study to our knowledge showed that Gd-enhanced MRI, especially DWI, depicted small liver metastases in approximately 10% of patients with a potentially resectable pancreatic cancer without liver metastases on CECT [26]. The reported sensitivity was 73–80% and the specificity 96–100%. However, due to the relatively low prevalence of patients with liver metastases in their study population, in total only 11 patients with liver metastases were included in this study.

In the DIA-PANC study we will determine the diagnostic accuracy of Gd-enhanced MRI with DWI in the detection of liver metastases in patients with all stages of PDAC.

## Methods

### Design

The DIA-PANC study is a prospective, international, multicenter, diagnostic cohort study investigating diffusion-weighted, Gd-enhanced MRI for the detection of liver metastases in patients with pancreatic cancer.

This protocol was written and reported according to the Standard Protocol Items: Recommendations for Interventional Trials (SPIRIT) Guidance and Checklist [27].

### Study population

All patients with (suspected) pancreatic ductal adenocarcinoma are eligible to be included in this study and will be actively recruited at the outpatient clinic by the treating physician. Written informed consent will be obtained by one of the members of the research team. We will include patients until 138 patients with liver metastasis are included, with a maximum total of 465 patients. Exclusion criteria are age below 18 years, previous treatment for pancreatic cancer, concomitant malignancies (except for adequately treated basocellular carcinoma of the skin, subjects with prior malignancies must be disease-free for at least 5 years), contraindications for MRI or CECT (i.e. untreatable contrast allergy, severe renal function impairment, not MRI compatible medical implants), insufficient command of the local language and pregnancy. This study has been approved by the ethical board of our university medical center. Approval of the local medical ethical board is obliged before the start of inclusion in the participating hospitals.

### Specific withdrawal of patients

Patients with adenocarcinoma of the distal common bile duct, papilla of Vater or duodenum, patients with a neuro-endocrine tumor or patients with benign tumors will be excluded from analysis and follow-up.

### Primary outcome

The sensitivity and specificity of Gd-enhanced MRI with DWI for the detection of liver metastases in patients with pancreatic cancer.

### Secondary outcomes

The secondary outcomes of this study are: sensitivity and specificity of CECT for the detection of liver metastases; sensitivity and specificity of MRI and CECT for the prediction of resectability; and the effect of the MRI on patient management.

### Data collection

All patients will be assigned a unique participant code. The key will be stored separately from the data. We plan to collect the following baseline data (age, sex, performance status (WHO performance score), American Society of Anesthesiologists physical status, body mass index, weight loss, decreased appetite, diabetes mellitus, previous liver or pancreatic diseases, smoking and alcohol status and tumor markers (CEA and CA19–9)) using the data management system Castor EDC (Castor Electronic Data Capture, Ciwit BV, Amsterdam, The Netherlands). Data on diagnostic procedures (like endoscopic imaging and biopsies), treatment and clinical follow-up will be collected during the entire study period by the local treating physicians or the trial coordinators using Castor EDC. Patients will be asked to fill in validated quality of life questionnaires (EORTC QLQ-C30 and QLQ-PAN26) at baseline and after 3-, 6- and 12-months follow-up.

### MRI and CT

**Fig. 1:**
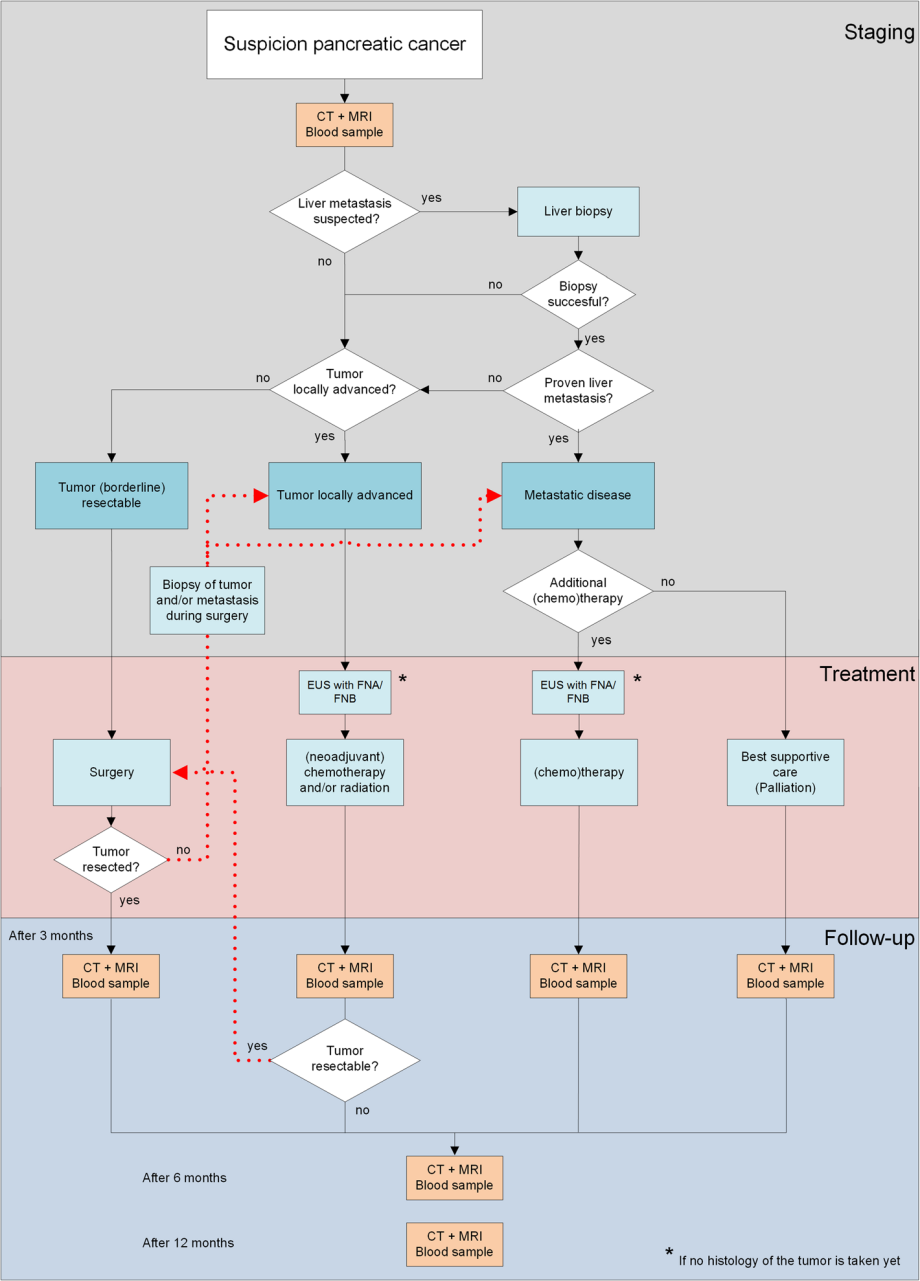
Flowchart of study schedule and procedures

MRI scans will be made on a 3 T scanner with T2 weighted imaging, using an intravenous gadolinium-based contrast agent with a T1 weighted pre-contrast, arterial and portal-venous phase, DWI with b-values of 50, 500 and 800 s/mm^2^ and with a Magnetic Resonance Cholangio-Pancreatography (MRCP). CECT scans are performed with intravenous iodine contrast agent with a pancreatic phase of the upper abdomen, a portal venous phase of the entire abdomen. Additionally, the chest will be staged using chest CT. MRI and CECT will be performed at baseline and after 3-, 6- and 12-months follow-up, the schedule is displayed in a flowchart in Fig. 1.

### Interpretation of MRI and CT

All MRI and CECT scans will initially be evaluated by the local radiologist and the findings will be included in the clinical decision making. The MRI and CECT scans will also be independently evaluated by a second radiologist blinded for findings of the first evaluation and the clinical outcome. If the MRI and CECT of one patient is evaluated by the same radiologist a minimum interval of 6 weeks will be used to minimize the risk of recall bias.

The MRI and CECT scans will be analyzed for local resectability and suspicious liver lesions. Number of liver lesions, lesion size, liver segment, presumed diagnosis of suspicious liver lesions (indeterminate or malignant) and imaging characteristics on MRI will be noted.

### Reference standard

Indeterminate or malignant liver lesions will be further investigated histopathologically. The first step in obtaining histological proof of suspected liver lesions on CECT and/or MRI is transabdominal ultrasound of the liver. Biopsy will be performed of visible liver lesions and analyzed with routine histological examination. When lesions are not visible or there is no histological proof of the visible lesions, the next step is surgical exploration (laparoscopic or open) in (borderline) resectable pancreatic cancer. In case liver lesions are identified a frozen section is performed. Hereafter, patients are treated according to standard care protocol.

For patients with suspected liver lesions without histopathological proof, follow-up imaging with paired CECT and MRI at 3, 6 and 12 months will serve as an alternative reference standard. Lesions that are growing or increasing in number over time will be considered metastases.

### Definitions

On MRI liver lesions are defined as malignant on DWI when they are (moderately) hyperintense at b = 50 s/mm^2^ and remains hyperintense at b = 800 s/mm^2^. A lesion is considered benign when it is hyperintense at b = 50 s/mm^2^ and shows a substantial decrease in signal intensity at higher b values (b = 500 and b = 800 s/mm^2^). If none of the criteria is met, a lesion is classified as indeterminate.

On CECT liver lesions are defined as malignant if they are hypodense, not showing typical features of a simple cyst (fluid attenuation measurements, round-oval, well-defined borders, no contrast enhancement), hemangioma (localization next to vessels, peripheral nodular enhancement, centripetal fill-in), or focal fatty infiltration (geographic hypodense area, angular margins, typical location). If a lesion is showing signs of simple cyst, hemangioma or focal fatty infiltration it is defined as benign. If a lesion is too small to characterize it is classified as indeterminate.

TNM status is classified according to the American Joint Committee on Cancer (AJCC, 8th edition) [28]. Lymph nodes are defined as suspicious if they are rounded and ≥ 5 mm or if they are not-rounded with the shortest axis ≥ 10 mm.

### Safety and ethics

There is a low risk and low burden for patients participating in this study. Patients might benefit from study participation due to possible improvement of detection of liver metastases. The contrast agent used for MRI has few known side effects and rarely leads to a severe allergic reaction [29]. Extra CECT scans might be performed in some study patients with the associated radiation and contrast exposure. Patients diagnosed with pancreatic cancer have a 5-year overall survival of 8%. Radiation-induced cancer has a latency period that substantially exceeds 5 years. Therefore, the health risk for this specific oncologic patient group is almost negligible.

MRI can lead to earlier detection of liver metastases, however in some patients these lesions might be too small to biopsy. Consequently, we cannot always provide the patient certainty about the nature of the liver lesions detected with MRI. Furthermore, in follow-up local recurrence or metastases might be detected before a patient has symptoms. This may be seen as a disadvantage by some individuals.

### Statistics

#### Sample size

The sample size for the study was calculated for the primary endpoint (sensitivity and specificity of MRI for the detection of liver metastases).

The sample size is calculated based on a method for power calculations for diagnostic studies described by Jones et al. [30]. Based on literature and our previously performed retrospective study [9, 31–35] we estimate the sensitivity of MRI will be approximately 90%. In literature the specificity for MRI is usually higher than the sensitivity, therefore we based our sample size calculation on the sensitivity only. With an expected sensitivity of 90%, confidence interval of 95% (Z = 1.96) and α = 0.05, 138 patients with metastasis are required for analysis. Based on literature the expected percentage of patients with liver metastases is approximately 40% [3, 36]. With an expected inclusion rate of 80% (assuming 20% cannot be analyzed optimally, e.g. because no representative liver biopsies could be acquired, mortality before first follow-up or withdrawal) we need approximately 433 patients. In case the proportion of patients with metastases is not equal to 40% in our cohort, we will include until we reach 138 patients with liver metastasis or up to a maximum total of 465 patients.

#### Analysis

Analysis will be done using SPSS (IBM Corp., Armonk, New York, USA). Continuous variables will be summarized with standard descriptive statistics including mean, standard deviation, median, and range. Categorical variables will be summarized with frequencies. A *p*-value less than 0.05 is considered statistically significant.

For the analysis of the diagnostic accuracy (sensitivity and specificity) a 2 × 2 cross tabulation will be made comparing MRI and CECT to histopathology and follow up. Performance of CECT and Gd-enhanced MRI with DWI will be compared using McNemar’s test. We will report the changes made in patient management in a descriptive manner. Median and 1-year survival will be reported. Survival endpoints (disease free survival and overall survival) will be analyzed using Kaplan-Meier plots. Survival curves are compared using the log rank test. We will compare the results of both readers to determine the inter-observer variability. A Cohen’s Kappa (k value) of 0.81–1.00 is interpreted as excellent, 0.61–0.80: substantial agreement, 0.41–0.60: moderate agreement, 0.21–0.40: fair agreement, and 0.00–0.20: poor agreement.

We partly anticipated missing data by introducing the composite reference standard of follow up. Unfortunately, missing data still can occur when, for instance, a patient suspected of having metastatic disease, does not have histopathological confirmation and dies before the composite reference standard follow up could take place. If necessary, additional analysis will be performed to determine the robustness of the results and to deal with missing data.

### Trial status

The first patient was included on December 21st 2017. At the time of protocol submission (July 23th 2020) active inclusion of patients has started in six centers; Radboud University Medical Center (Nijmegen, the Netherlands), Konstantopouleio General Hospital (Athens, Greece), Medisch Spectrum Twente (Enschede, The Netherlands) and Jeroen Bosch Hospital (Den Bosch, The Netherlands), University Medical Center Groningen (Groningen, The Netherlands), and University Hospital Ramón y Cajal (Madrid, Spain) and a total of 190 patients have been included. Four centers are preparing to start with inclusion; Inselspital Universitätsspital Bern (Bern, Switzerland), UCHealth University of Colorado Hospital (Denver, United States of America), Azienda Ospedaliera Universitaria Integrata Verona (Verona, Italy), and Policlinico A Gemelli (Rome, Italy). Inclusion of patients is expected to be finished December 2021.

## Discussion

The purpose of the DIA-PANC trial is to investigate the diagnostic accuracy of contrast-enhanced diffusion-weighted MRI in patients with suspected PDAC for the detection of liver metastases. Additionally, we will evaluate whether performing contrast-enhanced diffusion-weighted MRI will improve the detection of liver metastases compared to CECT by determining the sensitivity and specificity of CECT for the detection of liver metastases.

Despite the good diagnostic performance of MRI for liver metastases, the benefits of MRI remain unclear, mostly because of low level of evidence, heterogeneity, and bias in the performed studies. Two recently published meta-analyses have suggested the results should be confirmed by performing a well-designed and sufficiently powered study directly comparing liver CT and MRI in the same cohort [37, 38].

A major difficulty in the interpretation of the current literature is that most studies are retrospective often only reporting on a subset of patients actually undergoing a resection, patients with borderline resectable tumors, or patients with indeterminate liver lesions on CECT. These patients have a higher probability of having liver metastases. However, in an era of neoadjuvant therapy, local ablative therapy for advanced tumors, expensive targeted therapies, and resection of oligometastases, MRI may be beneficial to patients with all stages of PDAC. Therefore, all patients with suspected PDAC are eligible for inclusion in the DIA-PANC.

MRI field strength, 1.5 T versus 3 T, was a significant factor in the heterogeneity between studies that was found in a meta-analysis. 3 T MRI had a higher sensitivity (89%) and a lower specificity (88%) for diagnosing liver metastasis compared to 1.5 T MRI (sensitivity 80% and specificity 100%) [37]. Because the signal-to-noise ratio and the lesion-to-liver contrast are higher on 3 T MRI than on 1.5 T MRI, it is reasonable that a 3 T MRI permits a higher lesion detection rate [39, 40]. In the DIA-PANC study we plan to perform all MRIs on a 3 T scanner. A potential downside of a multicenter design is the intervendor variability that could occur when comparing the quantitative Apparent Diffusion Coefficient (ADC) value, this variability seems to be more pronounced at 3 T than at 1.5 T [41].

Availability of MRI is not expected to be an issue, as MRI is available in every expert center for pancreatic diseases. However, problems with MRI capacity could arise due to the need for MRI within a short interval after CT. A time interval of two weeks was chosen to provide a feasible time frame for MRI to be performed and no interval lesions are expected within this time interval [4].

The DIA-PANC trial is the first international prospective multicenter cohort study about the diagnostic accuracy of contrast-enhanced diffusion-weighted MRI. On the World Health Organization trial registry website (ICTRP), incorporating all (inter) national trial registries, there are only four other prospective trials registered in this field.

The first trial is a completed French prospective multicenter trial, presumably the only one prospective study that has been published [26]. The study has been performed in 118 patients with potentially resectable pancreatic cancer on a 1,5 T scanner using gadobenate dimeglumine (MultiHance) as contrast agent. The study has been performed to assess the diagnostic performance of diffusion-weighted MRI for the preoperative diagnosis of liver metastasis and the modification of therapeutic strategy as a consequence of the diagnosis of liver metastasis on diffusion-weighted MRI [42].

The second trial is a British single center observational study with a target sample size of 30 patients with confirmed or suspected pancreatic cancer referred for pancreaticoduodenectomy and is completed recently. The primary outcome of this study is the proportion of patients correctly identified by MRI to have lymph node, peritoneal, or liver metastases. To our knowledge, the results have not been published and there is no information on scan parameters and contrast agent available [43].

The third trial from Australia is the only randomized controlled trial. The study has a target sample size of 24 patients and is not yet recruiting. The aim of the study is to compare the 12-month recurrence rate in patients with locally operable pancreatic adenocarcinoma managed with standard preoperative assessment of liver metastases with CECT, versus preoperative assessment with liver specific contrast MRI [44].

The fourth trial is a Chinese comparative study and is not yet recruiting. The study aims to compare liver specific contrast MRI and CECT in liver metastasis of pancreatic cancer with a target sample size of 60 patients [45].

The DIA-PANC trial hypothesizes a superior value of MRI for the detection of liver metastases compared to CECT. To reliably determine the diagnostic accuracy the gold standard is histopathology of the liver lesions. Considering it is not always possible, and sometimes even unethical, to obtain histopathological proof of every lesion, follow-up is used as a reference standard. Hence, we are able to simultaneously gather information on (early) local recurrence or metastases after resection, disease progression, and therapy response evaluation on MRI and CECT.

In conclusion, the DIA-PANC trial is expected to report high-level evidence of the diagnostic accuracy of MRI for the detection of liver metastases compared to CECT, resulting in significant value for clinical decision making, guideline development and improved stratification for treatment strategies and future trials. Furthermore, DIA-PANC will contribute to our knowledge of liver metastases regarding incidence, imaging characteristics, their number and extent, and their change in time with or without treatment. When our hypothesis is confirmed, it will enhance the worldwide implementation of MRI and consequently improve personalized treatment of patients suspected of PDAC.

## Data Availability

The complete dataset will be property of the Sponsor, all participating institutions will own the dataset of the included patients from their center. Public access to the full trial protocol, trial-related documents, participant-level dataset, and statistical code may be made available on request.

## Abbreviations

ADC: Apparent Diffusion Coefficient
AJCC: American Joint Committee on Cancer
Castor EDC: Castor Electronic Data Capture
CEA: Carcinoembryonic Antigen
CECT: Contrast Enhanced Computed Tomography
CA19–9: Carbohydrate Antigen 19–9
DIA-PANC: Diagnostic accuracy of contrast-enhanced diffusion-weighted MRI for liver metastases of pancreatic cancer
DWI: Diffusion-Weighted Imaging
EORTC: European Organization for Research and Treatment
EUS: Endoscopic Ultrasound
FNA: Fine Needle Aspiration
FNB: Fine Needle Biopsy
Gd: Gadolinium
ICTRP: International Clinical Trials Registry Platform
MRCP: Magnetic Resonance Cholangio-Pancreatography
MRI: Magnetic Resonance Imaging
PDAC: Pancreatic ductal adenocarcinoma
SPSS: Statistical Package for the Social Sciences
TNM: Tumor, Node, Metastasis
WHO: World Health Organization
QLQ-C30: Quality of life questionnaire including 30 questions
QLQ-PAN26: Pancreatic cancer module of quality of life questionnaire including 26 questions
